# Low coverage of species constrains the use of DNA barcoding to assess mosquito biodiversity

**DOI:** 10.1038/s41598-024-58071-1

**Published:** 2024-03-28

**Authors:** Maurício Moraes Zenker, Tatiana Pineda Portella, Felipe Arley Costa Pessoa, Johan Bengtsson-Palme, Pedro Manoel Galetti

**Affiliations:** 1 Laboratório de Biodiversidade Molecular e Conservação, Departamento de Genética e Evolução, Universidade Federal de São Carlos, São Carlos, 13565-905 Brazil; 2 Departamento de Ecologia, Instituto de Biociências, Universidade de São Paulo, São Paulo, Brazil; 3 Laboratório de Ecologia de Doenças Transmissíveis na Amazônia, Instituto Leônidas e Maria Deane, Fiocruz Amazônia, Manaus, Brazil; 4 Division of Systems and Synthetic Biology, Department of Life Sciences, SciLifeLab, Chalmers University of Technology, 412 96 Gothenburg, Sweden; 5 Department of Infectious Diseases, Institute of Biomedicine, The Sahlgrenska Academy, University of Gothenburg, Guldhedsgatan 10A, 413 46 Gothenburg, Sweden; 6 Centre for Antibiotic Resistance Research (CARe), Gothenburg, Sweden

**Keywords:** Taxonomy, Entomology

## Abstract

Mosquitoes (Culicidae) represent the main vector insects globally, and they also inhabit many of the terrestrial and aquatic habitats of the world. DNA barcoding and metabarcoding are now widely used in both research and routine practices involving mosquitoes. However, these methodologies rely on information available in databases consisting of barcode sequences representing taxonomically identified voucher specimens. In this study, we assess the availability of public data for mosquitoes in the main online databases, focusing specifically on the two most widely used DNA barcoding markers in Culicidae: COI and ITS2. In addition, we test hypotheses on possible factors affecting species coverage (i.e., the percentage of species covered in the online databases) for COI in different countries and the occurrence of the DNA barcode gap for COI. Our findings showed differences in the data publicly available in the repositories, with a taxonomic or species coverage of 28.4–30.11% for COI in BOLD + GenBank, and 12.32% for ITS2 in GenBank. Oceanian, Afrotropical and Oriental biogeographic regions had the lowest coverages, while Nearctic, Neotropical and Palearctic had the highest. The Australian region had an intermediate coverage. In general, countries with a higher diversity of mosquitoes and higher numbers of medically important species had lower coverage. Moreover, countries with a higher number of endemic species tended to have a higher coverage. Although our DNA barcode gap analyses suggested that the species boundaries need to be revised in half of the mosquito species available in the databases, additional data must be gathered to confirm these results and to allow explaining the occurrence of the DNA barcode gap. We hope this study can help guide regional species inventories of mosquitoes and the completion of a publicly available reference library of DNA barcodes for all mosquito species.

## Introduction

Mosquitoes belong to the family Culicidae, and some of their species are notoriously known as vectors of malaria, various forms of filariasis etiological agents, and numerous arboviruses such as the dengue, yellow fever, and West Nile viruses, which can affect humans as well as wild animals^1,2^. In addition, mosquito feeding may also result in pathogen transmission between livestock reservoirs and, incidentally, humans; also, their bite can cause stress and pain in humans and livestock animals^3^. On the other hand, mosquitoes are important players in the aquatic and terrestrial food chains^4^, promoting nutrient recycling^5^ and pollination^6^. Therefore, a solid knowledge on the taxonomy of mosquitoes is fundamental to understanding their ecology, and to promoting their control.

Similarly to many insect taxa, the family Culicidae has a high species richness with a total of 3,570 recognized species^7^, which makes accurate species taxonomic identifications challenging. The identification of mosquito species has traditionally been done using morphological characters such as male genitalia and scales color pattern^8^, although other methods such as those employing pheromones^9^, infrared^10^, and even acoustic signals^11^, have also been employed. Among these non-traditional methods, the molecular methods of species identification, more specifically DNA barcoding^12^, have gained popularity in the last decades because they do not require direct assistance of highly in-demand, specialized taxonomists; also, they are fairly accurate, and their use has increased recently due to reduction in sequencing costs^13^. In addition, the recent development of next-generation sequencing, new protocols and bioinformatics tools have allowed for species identification in bulk mosquito samples^14^, and their detection in environmental samples^15,16^. However, to obtain a species name, all these methodologies rely on information available in databases, which allow for species identification of query sequences by sequence similarity^17^.

Since DNA barcodes started being widely used in mosquito species identification in the early 2000s, a large number of the mtDNA COI gene barcodes have been produced and, to a lesser extent, also barcodes of the ribosomal RNA genes, including—especially—the non-coding ITS2 (internal transcriber spacer region 2). However, despite many studies having demonstrated the usefulness of these markers for accurate mosquito species identification^18–20^, the availability of COI and ITS2 barcodes for Culicidae in the online databases remains to be evaluated. In addition, no study so far has tested hypotheses concerning factors that could explain taxonomic (or species) coverage and the occurrence of the DNA barcode gap with the whole data publicly available for Culicidae in the databases. The DNA barcode gap is here defined as a difference between minimum intraspecific identity and maximum interspecific identity equal or greater than zero (see Methods section). If the gap is present, it suggests that a taxonomic entity corresponding to a species can be differentiated from other species entities. The results of such a study could be very useful in guiding actions to produce new taxonomic and genomic data that can improve the identification of mosquitoes using barcodes.

Here we assess the availability of COI and ITS2 barcodes for mosquitoes with data obtained from the main barcode databases using APIs (Application Programming Interfaces^21^). We then use the available data to investigate different hypotheses on COI barcoding of mosquitoes. In particular, we address the following hypotheses regarding how well mosquito species in the biogeographic regions and countries of the world are covered in the analyzed data: (1) coverage is lower in the most species-rich and/or endemic-rich biogeographic regions; (2) coverage is lower in countries with higher species richness and/or endemic richness; (3) countries with a higher number of sequences also have a higher coverage; and (4) coverage is higher in countries with a higher number of medically important species. In addition, we also analyzed hypotheses with respect to the effectiveness of retrieving species-level taxonomic identifications of mosquitoes based on the occurrence of the DNA barcode gap: (1) a high percentage of mosquito species are yet to be represented in the databases; more effective species identification is related to whether a species (2) has a higher number of available sequences, (3) has a higher mean sequence length, (4) is medically relevant, (5) is represented by sequences in a higher number of countries, and (6) is covered by a higher number of countries within its known distribution in the analyzed database.

## Results

A total of 3570 species occurring in 317 countries throughout the world were compiled from the Culicidae taxonomic catalogue (Supplementary Data S1). The queries performed for species, individually, in BOLD, resulted in a data set with 59114 rows (records) and 80 columns, including 1054 species, which represents 29.52% of all species of Culicidae, although sequences were unavailable for 40 species (Table 1). A total of 21 different markers were represented in the data set, most of them in less than 40 rows (Supplementary Table S1). No species name was present exclusively in the data set including markers other than COI-5P. In 5145 rows, the gene region and sequence were unavailable and, after excluding these and including only rows identified as COI-5P, 50127 rows remained. In addition, the country name was unavailable for 5860 rows and, in 10 rows, the country name was misidentified. Among the rows without a country name, 5813 had neither latitude nor longitude, and this information was questionable in the remaining rows, and thus these were excluded, resulting in a data set with 44257 rows. This data set included a total of 945 species. However, for 15 of the species, the number of countries where the species are mentioned in BOLD exceeded the number of countries referring to the species in the taxonomic catalogue. Therefore, these species were excluded, resulting in a final data set with 43796 sequences and 930 species, hereafter called BS_01 (Table 1, Supplementary Data S2).

**Table 1:** Number of records of barcode sequences (COI and ITS2) and mosquito species publicly available in BOLD and GenBank according to queries made through R interfaces to BOLD and NCBI's EUtils.

	COI (BOLD)	COI (unique to BOLD)	COI (GenBank)	COI (unique to GenBank)	COI (shared between BOLD and GenBank)	COI total (BOLD and GenBank)	ITS2 (GenBank)
No of species^1^	3570	–	–	–	–	–	–
No of sequences^2^	59114	21645^ g^	45232	7927	37545f.	67117	13347
No of species^2^	1054^b^	101^c^	1008^a^	55	953	1109^d^	440^e^
No of sequences^3^	43796	–	–	–	–	–	–
No of species^3^	930	–	–	–	–	–	–

^1^Number of species compiled from the taxonomic catalogue (Wilkerson et al.^7^).

^2^According to data sets obtained in queries made with species names compiled from the taxonomic catalogue.

^3^Same as in ^2^ but after filtering out all genes, except for COI, and rows (records) in which either gene name or country name was unavailable or misidentified; a total 15 species corresponding to 461 sequence records were also filtered out because the number of countries referred to them in BOLD was higher than in the taxonomic catalogue. The number of species reported in ^2^ and ^3^ are based on species names that matched exactly those compiled from the taxonomic catalogue.

^a^After excluding *Culex fuscanus* which is not included in the species list compiled from the taxonomic catalogue.

^b^This number is reduced to 1014 if 40 species without sequences are excluded.

^c^This number is reduced to 67 if 34 species without sequences are excluded.

^d^This number is reduced to 1075 if 34 species without sequences are excluded.

^e^After excluding 8 species which are not included in the taxonomic catalogue.

^f^This number is reduced to 37469 or 37305 sequences if shared duplicated accession numbers present in one data set but absent in another are excluded.

^g^This number is reduced to 21076 if 569 records mined from GenBank are excluded (see text for details).

The queries of COI performed for the species, individually, on GenBank, resulted in a file with 45232 rows and two columns, one for the sequence data and another for the sequence itself. A total of 1009 species were present in this data set and, after comparing the species names with those from the taxonomic catalogue, 1008 species remained (Table 1, Supplementary Data S3). However, sequences were unavailable for 34 species unique to the BOLD, and a total of 567 records unique to BOLD were in fact mined from GenBank. BOLD and GenBank shared a total of 37545 records and 953 species, and the total number of GenBank records and species present in BOLD + GenBank was 67117 and 1109, respectively. This number corresponds to 31.06% of all species in the taxonomic catalogue, although sequences were missing in 34 species (Table 1). The queries of ITS2 performed for the species, individually, on GenBank, resulted in a file with 13943 rows and two columns and, after filtering out all sequence names not related to ITS2, 13347 rows remained (Table 1). A total of 448 species was found in this data set, including eight species not included in the taxonomic catalogue (Supplementary Data S3). After removing these, 440 species remained (Table 1), corresponding to 12.32% of all Culicidae species. In 409 species, COI and ITS2 barcodes were available, whereas only ITS2 barcodes were available for 31 species.

**Figure 1:**
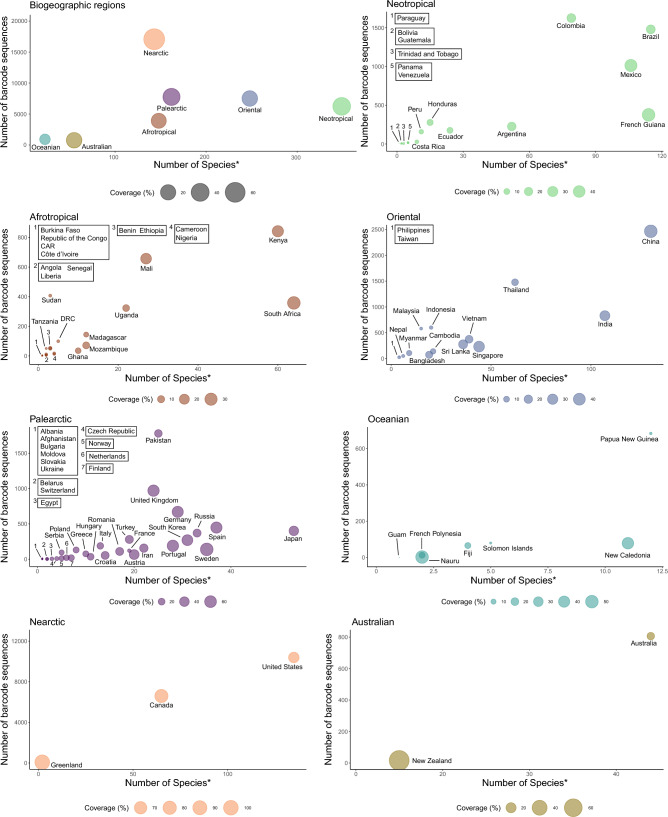
Taxonomic coverage of mosquitoes in biogeographic regions and countries of the world. To facilitate reading, only countries with 100 or more species recorded in Wilkerson et al.^7^ were included in the figure for Neotropical, Afrotropical and Oriental regions, and countries with 60 or more species recorded for the Palearctic regions. The x and y axis refer to the number of mosquito species and their COI barcode sequences publicly available in BOLD, respectively. The number next to each rectangle and bubble refers to the number of species in the database for the country/countries inside the rectangle. *Note that incomplete names of species or species names with additional names other than those reported in Wilkerson et al.^7^ were not used in this figure. See text for details.

Figure 1 depicts the taxonomic coverage of mosquito species in 95 out of 142 countries and seven biogeographic regions for which data was available in BOLD, plotted against the number of barcode sequences and the number of species available to each country and biogeographic region. A complete list with taxonomic coverages and other analyzed variables for all countries and biogeographic regions is available in Supplementary Data S4. Oceanian (5.67%), Afrotropical (16.89%) and Oriental (19.6%) biogeographic regions had the lowest taxonomic coverage, while Nearctic (64.7%), Neotropical (34.15%) and Palearctic (29.29%) had the highest (Fig. 1). The Australian region had an intermediate taxonomic coverage (20.89%). According to data on species richness and the percentage of endemic species compiled from the taxonomic catalogue, the Neotropical, Oriental and Afrotropical regions have the highest species richness and percentages of endemic species, which partially corroborates our hypothesis that the most species-rich and endemic-rich regions are those with the lowest taxonomic coverage. Some of the countries with taxonomic coverages of mosquito species below 5% are Papua New Guinea, Philippines, Panama, Venezuela, Malaysia, and Indonesia—which, according to the taxonomic catalogue, have between 250 and 500 species recorded. The countries with taxonomic coverages of 50% or higher are, in most cases, high-income countries, especially from Europe and North America (Fig. 1).

The result of the VIF analysis did not show collinearity between the predictor variables used to explain taxonomic coverage (all predictor variables < 3, Supplementary Table S2). The model with all variables and with biogeographic regions as a random effect had the best fit (AIC = 1998.4). This and the other tested models are available in Supplementary Table S2, and the results of the best model to explain taxonomic coverage are available in Table 2. These results corroborate our hypotheses that countries with a higher number of sequences also have a higher taxonomic coverage, and that coverage is lower in countries with higher species richness (Table 2). However, our results refute the hypotheses that coverage is higher in countries with a lower number of endemic species, and that countries with a higher number of medically important species have a higher coverage (Table 2).

**Table 2:** Results of the two best models.

Model	Covariable	Coefficient	Std. error	Odds ratio	CI (95%)	P value
Taxonomic coverage	No of species	− 0.29	0.04	0.75	0.69–0.81	< 0.001
	No of sequences	0.78	0.05	2.18	1.98–2.39	< 0.001
	No of endemic species	0.10	0.03	1.10	1.04–1.16	< 0.001
	No of medically important species	− 0.27	0.04	0.76	0.70–0.83	< 0.001
Barcode gap	No of countries where the species is present in the database	− 1.39	0.29	0.25	0.14–0.44	< 0.001
	No of sequences	− 4.55	0.76	0.01	0.002–0.04	< 0.001

Taxonomic coverage (GLMM): best model explaining the percentage representing the number of mosquito species recorded in 142 countries available in BOLD, compared to the number of mosquito species with records in 317 countries and localities in the taxonomic catalogue (Wilkerson et al.^7^). Barcode gap (GLM): best model explaining the occurrence of the barcode gap. See text for details.

**Figure 2:**
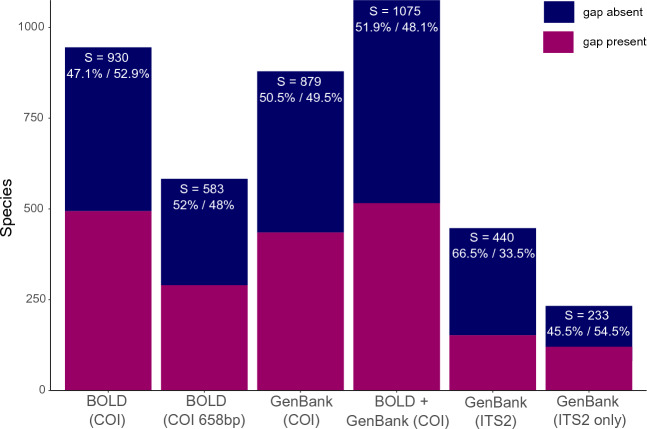
Proportion of barcode gap presence/absence in different data sets obtained from BOLD and GenBank, and number of species in the referred data sets. The bar identified as BOLD (COI) corresponds to the data set used in the statistical analyses (i.e., data set BS_01). The bar identified as BOLD (COI 658 bp) was produced based on the data set BS_01 and includes only standard barcode sequences of 658 bp in length. The bar identified as GenBank (COI) corresponds to a data set obtained from GenBank with a species composition that matched BS_01. The bar identified as BOLD (COI) + GenBank (COI) corresponds to a data set assembled with shared publicly available records between BOLD (COI) and GenBank (COI) as well as publicly available unique records to these databases. The bar identified as GenBank (ITS2) corresponds to a data set obtained from GenBank for ITS2 region, including flanking regions (5.8S and 28S), whereas the bar identified as GenBank (ITS2 only) includes ITS2 only. See text for details.

The results of the BLAST analyses for seven different data sets are depicted in Fig. 2 and in Supplementary Data S3, along with other analyzed variables for individual species used in the statistical tests. The gap was present in 52.9% of the 930 species included in the data set used in the statistical analyses (BS_01). In the remaining data sets for COI, the presence of the gap varied from 48 to 49.5%. Finally, in the two data sets for ITS2 obtained from GenBank, the presence of the barcode gap varied depending on whether the boundaries of ITS2 were determined. In the full data set, based on a file with 13347 sequences, where 5.8S and 28S may also be included, the barcode gap was present in 33.5% of 440 species. Differently, in the smaller data set based on a file with 5154 sequences and 233 species, where ITS2 was separated from its flanking regions, the gap was present in 54.5% of the species (Fig. 2).

The result of the VIF analysis did not show collinearity between the analyzed variables used to explain the presence of the DNA barcode gap (all predictor variables < 3, Supplementary Table S2). Based on AIC values, the models m_1 and m_2 did not have substantial fit differences in relation to model M_04 (AIC = 1070). However, we chose model M_04 because the additional variables included in the other models were not significant. All tested models and their results are available in Supplementary Table S2, and the results of model m_4 are available in Table 2. Our results showed that the lower the number of sequences available for a species, the higher the occurrence of the barcode gap; and that the lower the number of countries where a species is present in the database, the higher the occurrence of the barcode gap (Table 2). Therefore, these results, and the fact that the additional variables are not included in the best model, refute our initial hypotheses regarding the occurrence of the DNA barcode gap in the species with publicly available data.

## Discussion

To our knowledge, this is the first comprehensive and taxonomically guided accounting for COI and ITS2 barcodes, specifically for Culicidae, publicly available in BOLD and GenBank, and this is also the first attempt to explain species (taxonomic) coverage and the presence of the DNA barcode gap for mosquitoes on a global scale. The total number of species with COI sequences publicly available in BOLD and GenBank, in Table 1, confirms our initial hypothesis that a high percentage of mosquito species are yet to be represented in the databases and, similarly to many taxa, highlights the great deficit of data that can be used to assign species taxonomic names to query sequences^22^. This even includes one species (*Aedes fijiensis*) among 128 species referred to as medically relevant by Wilkerson et al.^7^, which is covered in neither BOLD nor GenBank. As for ITS2, the number of uncovered species is much lower, and those which are covered mostly overlap with COI.

A thorough analysis of the data used to produce the results in Table 1 revealed discrepancies in the diversity of data between BOLD and GenBank—which can be expected, given the different nature of both repositories^23,24^ and the different packages/APIs and their settings used to download the data. The file obtained with the package Bold consists of an 80-column table containing all available information concerning specimens and sequences, including collection locality for most of the records. Although the package rentrez allows access to several NCBI databases and a wide array of data types, the data relevant to this study that could be retrieved with this package was limited to accession numbers, record titles, and sequences—which are available, respectively, in the annotation fields VERSION, DEFINITION and ORIGIN on the NCBI nucleotide webpage of each record used in this study. In theory, the data on the specimens' collection localities could be retrieved with the package rentrez using the argument FKEY (feature annotated on sequence), which is supposed to allow the search for records of countries and other data types available in the annotation field FEATURES^25^. However, our tests performed on NCBI's nucleotide database with this argument failed, even when using the name of a country where the query species is known to occur and to be represented in that database.

The organization and completeness of the data obtained from BOLD using the Bold package facilitate direct and rapid access to mosquito species distribution; however, despite the overall completeness of our dataset from BOLD, many records lacked sequences and information on the specimens' collection localities. This is probably related to the fact that the data available in this database was obtained, independently, by a large number of institutions around the world, and some of them uploaded files with missing data to BOLD—inadvertently or not. Interestingly, all records without sequences (i.e., 5145 records) were unique to BOLD and, in nearly half of these records, country-related information was available (e.g., French Guina alone was mentioned in a total of 1979 records). In addition, a total of 40 species were exclusively present in the records without sequences unique to BOLD, although six of them were also available in GenBank (Table 1). It is also worth mentioning that 569 sequence records, among the records that were supposed to be unique to BOLD, had in fact been mined from GenBank^26^. In most of these records, the species name had been updated in BOLD, whereas in GenBank it remained unaltered or misspelled, which prevented rentrez from detecting and downloading the records. Although there is only a small difference between the species composition with records of COI in BOLD and GenBank (Table 1), it is very important to mention that the databases do not share exactly the same sequences. This must be considered when preparing a database for comparing sequences of mosquitoes obtained from next generation sequencing. Therefore, in this case, we recommend building a database using data downloaded from both BOLD and GenBank, which can be accomplished using dedicated R packages such as refdb^27^.

Another intriguing feature of our data set downloaded from BOLD was the presence of a relatively large number of “markers”, which includes an array of 21 genetic markers. Although the function “bold_seqspec” includes a query parameter “markercode”^28^, we assumed that this would allow the search for barcodes other than COI used for plants, fungi, and other organisms, since BOLD also covers these taxa^23^. However, similarly to the missing data for sequences and specimens' collection localities, this might be explained by the collaborative nature of this database, and by attempts of different research groups to use markers other than COI^18–20^.

In our GLMM analysis, the model that best explained the taxonomic coverage of countries included all four analyzed predictable variables. As expected, the higher the number of sequences in a country, the higher the taxonomic coverage of the country (Table 2), which implies a generally low variation in the number of sequences for each species in our data set downloaded from BOLD. In fact, approximately 62% of the 930 analyzed species had 1–10 sequences, and only six species had more than 1000 sequences (Supplementary Data S3). The species with more than 1000 sequences are exclusively those with a wide distribution and of high significance to the public health, such as *Aedes albopictus* and *Culex quinquefasciatus*^7^. Thus, one might expect a higher number of sequences obtained from specimens collected from a wide array of countries and different localities within each country.

The taxonomic coverage calculated for biogeographic regions (Fig. 1, Supplementary Data S4) and the data on species richness and percentage of endemic species for these regions, compiled from the taxonomic catalogue, partially confirm our hypotheses that coverage is lower in biogeographic regions with higher species richness and higher percentage of endemic species. However, to our surprise, the Neotropical region had a higher coverage than the Australian, even though species richness and percentage of endemic species are higher in the Neotropical region. It is clear, in Fig. 1, that Australia has a very small taxonomic coverage compared to the five Neotropical countries with the highest coverage—which is unexpected, considering that taxonomic research in general is well developed in Australia^29^. Therefore, based on our assessment of the data publicly available on BOLD, the mosquitoes of Australia should be considered a priority in barcoding projects, especially the endemic species, since data compiled from the catalogue showed that 114 species are endemic to Australia, while only 44 species are available for Australia on BOLD. A similar scenario is found among many low-income countries from the Afrotropical, Neotropical, Oriental and Oceanian regions with taxonomic coverages below 5% and at least 100 species referred to in the taxonomic catalogue. In most cases, this might be generally ascribed to low investment by local governments in taxonomy, despite the efforts of local scientists and/or well-funded research institutions located in high-income countries^30,31^. Interestingly, France showed a very low taxonomic coverage, which can be explained by the fact that two intensively surveyed and highly species-rich territories of France in the Afrotropical region without sequences in BOLD (Glorioso and Juan de Nova islands) were added along with Corsica and continental France to the taxonomic catalogue. This is not the case with the French Guiana and Guadeloupe—which are also French territories but have sequences in BOLD, showing a relatively high taxonomic coverage (Fig. 1, Supplementary Data S4) due to previous research done mainly by French scientists^32^. Although coverage values are below 50% in a few high-income countries, a vast majority of countries with a taxonomic coverage higher than 50% is comprised of high-income countries (Fig. 1, Supplementary Data S4). This can be explained by the resources available in these countries in terms of research funding and numbers of qualified researchers, and due to barcoding campaigns at the national level^33^.

Not surprisingly, our analyses showed that the higher the species richness in a country, the lower the taxonomic coverage of the country. However, it also showed that the higher the number of endemic species of a country, the higher the taxonomic coverage (Table 2). Because the country species records of a previous study showed that 50% of mosquito species are endemic^34^, we initially hypothesized that taxonomic coverage would be lower in countries with a higher number of endemic species. This proved untrue, probably because, based on the taxonomic catalogue, approximately 44% of the countries in our data set downloaded from BOLD did not include endemic species, and *ca.* 47% of countries with endemic species had a taxonomic coverage of 10% or higher (Supplementary Data S4). However, it is important to note that this result does not mean that the endemic species are well covered, but simply that countries with a higher taxonomic coverage tend to have a higher number of endemic species. Finally, our analyses showed that the lower the number of medically important species referred to a country, the higher its taxonomic coverage (Table 2). Our initial hypothesis was that countries with a higher number of medically important species would have invested more in the taxonomic research of mosquitoes, and thus would have a higher taxonomic coverage. Therefore, our result suggests that species of medical importance should be targeted in barcoding projects developed in countries with a lower taxonomic coverage, which corresponds largely to low-income countries. Although most mosquito species of medical importance are covered in the databases, the inclusion of additional records of already covered species would help to determine intra and interspecific genetic diversity, as shown elsewhere^35^.

The results of the BLAST analyses of COI barcodes showed that, in approximately 50% of the species available in the databases, a DNA barcode gap was absent (Fig. 2). In addition, a quick analysis of COI barcodes available on BOLD and made for Culicidae with the app BAGS^36^ showed that approximately 50% of the species shared a BIN (Barcode Index Number^37^), which confirms our results. This may be initially interpreted as ineffectiveness of COI barcoding in retrieving species taxonomic identifications^38^. Similar results and interpretation can also be extended to ITS2 barcodes, although this was achieved with a much smaller data set made of sequences that could be separated from their highly conserved flanking regions. However, it is important to stress that these results must be interpreted with caution, because several factors might have led to the absence of the DNA barcode gap without necessarily implying its ineffectiveness as a tool to identify species. First, it is important to mention that our barcode gap analysis approach employed a heuristic method (i.e., BLAST^39^) instead of a method based on genetic distances using a model of DNA substitution, such as that employed in BOLD^23^. Although other methods may be used to build a distance matrix with such a large data set, we chose BLAST because it is a very common tool to determine sequence similarity (and homology), and also because its results are easily reproducible. Another factor that might have led to the absence of a gap is the possible presence of nuclear mitochondrial pseudogenes (i.e. NUMTs), which causes a species to split even though this splitting is not based on homologous sequences, as recently shown elsewhere for insects in general^40^. An approach was described in Hebert et al.^40^ to detect and remove NUMTs, but this would require a different analytical approach than the one employed here, as well as a reduction in our data set size. In addition, the gap might be absent when the average divergence time of COI and the average divergence time of species differ from each other (i.e., incomplete lineage sorting,), or due to mating between different species and their hybrids (i.e., introgressive hybridization), as shown elsewhere^41^. However, perhaps the most common cause of the absence of the barcode gap is related to human errors^42^. It is possible, for example, that barcoded specimens that are supposed to belong to different species belong, in fact, to the same, or there might be cryptic species. Therefore, it is likely that the taxonomic boundaries of the species here analyzed have changed (or should be changed) in light of new evidence obtained with barcodes and integrative taxonomy^43,44^. Although this was not the aim of this study, we were able to cross check the presence of the barcode gap in the species shared between the file containing 233 species, in which ITS2 was separated from its flanking regions, and the file BS_01 used in the statistical analyses (Fig. 2). Among 206 shared species, ITS2 showed the presence of the gap in 112 species, while COI showed the gap in 74 species. In either COI or ITS2, the gap was present in 66.2% of the species. This strongly supports the notion that a multi-marker approach is better than using a single marker to determine the presence of the DNA barcode gap in mosquitoes.

Considering the arguments described above, it is important to use caution when interpreting the results of our GLM analysis, treating the presence of the barcode gap as a response variable. Although all our initial hypotheses were refuted and our best model showed that the lower the number of sequences of a species and countries in which a species occur, the higher the occurrence of the barcode gap, these results probably have technical explanations. Because the occurrence of the barcode gap could not be determined properly, additional data must be gathered to test our initial hypotheses regarding the factors influencing the presence of the DNA barcode gap. This is particularly true if we consider the results of recent studies suggesting that a much larger sampling effort of COI barcodes may be needed to capture intra and interspecific variation^35^.

The Culicidae is the most important group of insects for public health, and it is also relevant in agriculture and in the ecological balance of many terrestrial and aquatic habitats. However, as shown in this study, most mosquito species remain without any publicly available data that can be used in species identification using COI and ITS2 barcodes. Although data of additional species might have been uploaded to the analyzed databases since we downloaded the data sets used in this study, which is the case of *Aedes kochi*—one of the two species lacking COI and ITS2 barcodes among 128 species referred to as medically important (see Supplementary Data S3)—this is the most up-to-date and comprehensive report on DNA barcodes publicly available for mosquitoes online. Our analyses revealed that certain biogeographic regions and countries have a higher taxonomic coverage than others, which is probably related to the low investment by local governments in taxonomic research. These findings are relevant to guide the efforts of research groups and governmental agencies in developing mosquito species inventories in different parts of the world. Finally, the high number of species without a DNA barcode gap found using the two analyzed genetic markers revealed potential cryptic diversity in half of all known mosquito species, although additional data must be gathered to confirm these results, and to test the hypotheses initially proposed in this study. As a closing remark, we would like to advocate in favor of a better curatorship of voucher specimens representing sequences in the databases. Ideally, these vouchers should be identified based on comparisons with a type specimen and the employment of an integrative taxonomic approach that combines various genetic markers with morphological analyses. For mosquitoes, in particular, this will allow a better employment of DNA barcoding/metabarcoding in a diverse array of applications, including vector species detection and biodiversity monitoring.

## Methods

### Mosquito taxonomic data and distribution

The taxonomic names used in this study were obtained from the book *Mosquitoes of the World*^7^, which is the most comprehensive and up-to-date source of taxonomic data for the family Culicidae worldwide. The pages included in the taxonomic catalogue in the second volume of the book were imported into R with the help of the pdftools package^45^, and data was organized using various R packages, such as dplyr and tidyr^46,47^. Species names highlighted in bold and not indented, as defined by the authors as in compliance with the rules of the International Code of Zoological Nomenclature^48^ to represent species, as well as the names of the localities where the species are reported to were compiled. No species names reported as synonyms were compiled.

### Data sources and query options

The automated species identification of mosquitoes is routinely done using barcode sequences of COI and/or ITS2, and thus we used BOLD (https://www.boldsystems.org/) and GenBank (https://www.ncbi.nlm.nih.gov/nucleotide) to assess data on these markers. Both databases include COI sequences but, while GenBank includes COI and ITS2, the vast majority of sequences in BOLD correspond to COI barcode sequences. Additionally, BOLD also includes valuable data on the distribution of the specimens that were used as sources of DNA to generate the sequences, which is easily accessible via API. In contrast, many records of mosquitoes on GenBank do not include country of occurrence, and it can be difficult to access the collection data of the specimens via API; thus, the distribution of the specimens/taxa with barcode sequences reported/analyzed in this study is based exclusively on data obtained from BOLD.

To download data from BOLD, we used an approach based on mosquito species names currently in use. Since the species names compiled by Wilkerson et al.^7^ are the most authoritative taxonomic species names, we performed a query for each species separately, using the name of the species as the taxon argument. The queries were performed in July 2023 using the bold package (API)^28^ and using the function “bold_seqspec”, which retrieves both a specimen's data and its sequence. All data downloaded was verified to include COI sequences only, and all records without a sequence and a referenced country were removed. This was necessary to determine which countries had barcode sequences for the species represented in the database.

Two data sets were obtained from GenBank through NCBI's nucleotide database in August 2023—one for COI, and another for ITS2. The data sets were downloaded using the rentrez package (API)^25^ and, as described above, a query was performed for each species name compiled from the taxonomic catalogue, using the “ORGANISM” argument. The functions “entrez_search” and “entrez_fetch” were used to search for and download the data, respectively. In addition, we used COI, COXI and others (see Supplementary Table S3) as the “GENE” argument in the query for cytochrome oxidase subunit one. Since the internal transcriber spacer region 2 is not a coding region, we used ITS2, ITSII and others (see Supplementary Table S3) as “ALL” arguments. The query results for ITS2, including or not the flanking regions 5.8S and 28S, were further filtered to assure that the sequence names included any acronym or name representing the internal transcriber spacer region 2. The sequence names in the data sets were reorganized to include “GenBank accession number” and “taxonomy information” as separate columns, and these were compared to those downloaded from BOLD.

### Analyzed variables

To test our hypotheses concerning the possible factors affecting the coverage and effectiveness of the data publicly available on BOLD in delivering species taxonomic identification, we used two different metrics as response variables. First, we calculated the taxonomic coverage, defined as a percentage representing the number of species in the data set (i.e., BOLD) recorded in each country/biogeographic region compared to the number of species with records for each country/biogeographic region in the taxonomic catalogue (i.e., Wilkerson et al.^7^). To explain the pattern of taxonomic coverage found for the countries, we selected four explanatory variables: (1) number of species, (2) number of endemic species, and (3) number of medically important species recorded in each country as reported in the taxonomic catalogue; and (4) number of COI barcode sequences available for each country, obtained from BOLD, in our data set.

Secondly, we calculated the occurrence of the DNA barcode gap^49^ for all analyzed species and used it as a response variable. The DNA barcode gap is here defined as the difference between minimum intraspecific identity and maximum interspecific identity. If this difference is greater than zero, the gap is present, and if it is below or equal to zero, the gap is absent. However, many species are represented in BOLD by one sequence only. In these cases, the gap absence was considered detected if there was a total match between the sequence representing the species and sequences representing any other mosquito species (i.e., interspecific identity of 100%). For the species with more than one sequence, we did not establish any threshold percentage because a recent study showed that this percentage can vary according to the taxon analyzed^35^. We chose five explanatory variables to explain the occurrence of the barcode gap: (1) number of countries where a species is present in our data set obtained from BOLD; (2) geographic coverage, defined as the proportion of countries where a species is present in our data set obtained from BOLD, compared to the known distribution of that species recorded in the taxonomic catalogue; (3) whether a species is medically relevant or not (0 = not medically relevant, 1 = medically relevant), consistent with Wilkerson et al.^7^; (4) number of sequences available; and (5) average sequence lengths for the species in our data set obtained from BOLD.

### Comparisons with databases

The presence of the barcode gap was calculated for the data sets obtained from BOLD and GenBank using stand-alone BLAST analyses^50^. The algorithm used in this program has been widely employed to determine the identity of sequences reported as percentages for more than 20 years, and it has also been continuously improved^51,52^. We chose this method for comparing sequences because it is relatively fast, capable of processing a large number of sequences and taxa, and its results can be easily reproduced due to its wide availability. The BLAST program was installed on a personal computer and the data sets obtained as described above were converted to FASTA format and then used to build databases. The same FASTA files used to build the databases were also used as query files, and the first one hundred sequences with the highest percentage of identity compared to the queried sequence were kept. The results of the BLAST analyses were saved in CSV format, and the maximum interspecific identity and minimum intraspecific identity for each species were filtered. In order to compare the results obtained from the analyses above, we performed the same analyses with the following data sets: (1) standard COI barcode sequences (i.e., 658 bp) obtained from BOLD; and (2) a data set of ITS2 sequences, excluding 5.8S and 28S flanking regions, obtained from GenBank and processed with the help of the ITSx software^53^.

### Statistical analyses

To test our hypotheses regarding the available data for countries in BOLD, we fitted a generalized linear mixed-effects model (GLMM) with a Binomial family error distribution, having the taxonomic coverage of countries as response variable, and using, as predictors, the number of species, the number of endemic species, the number of medically important species, and the number of sequences. We chose GLMM because preliminary analyses showed that the model with the best fit had biogeographic regions as a random effect. In addition, to test our hypotheses regarding the available data for species in BOLD, we fitted a GLM (generalized linear model) with a Binomial family error distribution, having the presence of the barcode gap as response variable, and the following predictors: the number of countries where a species is present in our data set obtained from BOLD, geographic coverage, the identification of the species as medically relevant or not, the number of sequences available, and the average sequence length for the species. To evaluate which variables were more suitable to explain the response variables, we fitted additive models with all possible combinations of explanatory variables. Then, we calculated the Akaike Information Criterion (AIC) for each model from their log-likelihoods, number of parameters, and sample size. The model with the lowest AIC was considered the best among the candidates, whereas those with ΔAIC < 2 were considered equally plausible^54^. Before the model selection, we performed a variance inflation factor (VIF) analysis to test for multicollinearity between variables, which represents the amount of variability of a covariate explained by other covariates. All variables with a VIF value lower than 5 were excluded from the analyses, as suggested elsewhere^55^.

## Supplementary Information

Supplementary Information 1.

Supplementary Information 2.

Supplementary Information 3.

Supplementary Information 4.

Supplementary Table 1.

Supplementary Table 2.

Supplementary Table 3.

## Data Availability

The data set used in the statistical analyses performed in this study is available in Supplementary Information S2 and S4. The remaining data sets are available from the corresponding author upon reasonable request.
