# Correction: Low coverage of species constrains the use of DNA barcoding to assess mosquito biodiversity

**DOI:** 10.1038/s41598-025-02631-6

**Published:** 2025-06-11

**Authors:** Maurício Moraes Zenker, Tatiana Pineda Portella, Felipe Arley Costa Pessoa, Johan Bengtsson-Palme, Pedro Manoel Galetti

**Affiliations:** 1 Laboratório de Biodiversidade Molecular e Conservação, Departamento de Genética e Evolução, Universidade Federal de São Carlos, São Carlos, 13565-905 Brazil; 2 Departamento de Ecologia, Instituto de Biociências, Universidade de São Paulo, São Paulo, Brazil; 3 Laboratório de Ecologia de Doenças Transmissíveis na Amazônia, Instituto Leônidas e Maria Deane, Fiocruz Amazônia, Manaus, Brazil; 4 Division of Systems and Synthetic Biology, Department of Life Sciences, SciLifeLab, Chalmers University of Technology, 412 96 Gothenburg, Sweden; 5 Department of Infectious Diseases, Institute of Biomedicine, The Sahlgrenska Academy, University of Gothenburg, Guldhedsgatan 10A, 413 46 Gothenburg, Sweden; 6 Centre for Antibiotic Resistance Research (CARe), Gothenburg, Sweden

Correction to: *Scientific Reports* 10.1038/s41598-024-58071-1, published online 28 March 2024

The original version of this Article contained errors. In biogeographyAnalysisUtD (the file used to produce the figure comparing biogeographic regions in Fig. 1), the data was duplicated for some countries and there were copies of the same data for a country in two biogeographic regions. Since the file biogeographyAnalysisUtD was not used in the statistical analyses, all the results remain valid.

The original Figure 1 and its accompanying legend appear below.

**Fig. 1:**
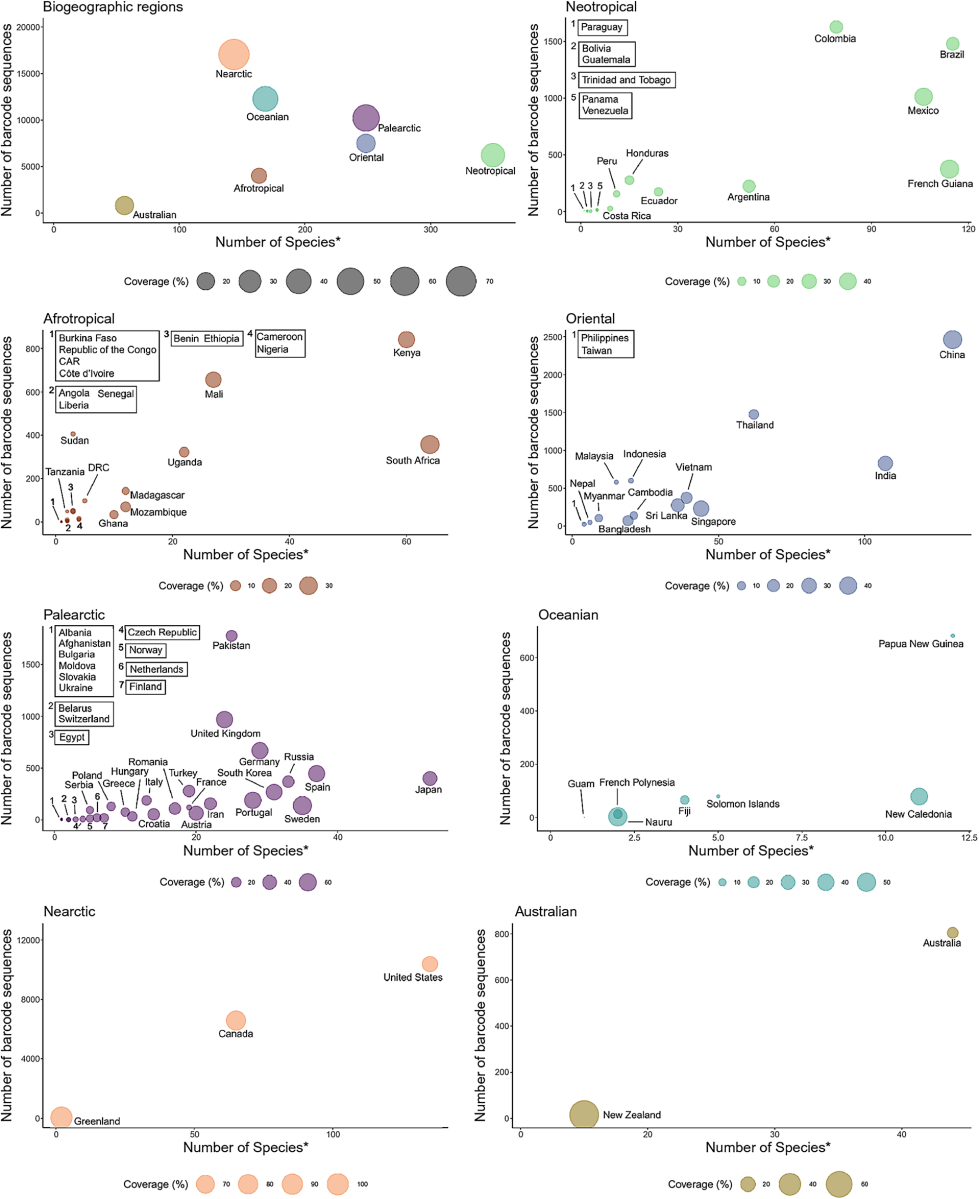
Taxonomic coverage of mosquitoes in biogeographic regions and countries of the world. To facilitate reading, only countries with 100 or more species recorded in Wilkerson et al.^7^ were included in the figure for Neotropical, Afrotropical and Oriental regions, and countries with 60 or more species recorded for the Palearctic regions. The x and y axis refer to the number of mosquito species and their COI barcode sequences publicly available in BOLD, respectively. The number next to each rectangle and bubble refers to the number of species in the database for the country/countries inside the rectangle. *Note that incomplete names of species or species names with additional names other than those reported in Wilkerson et al.^7^ were not used in this figure. See text for details.

As the result of this error, in the Abstract,

“Afrotropical, Australian and Oriental biogeographic regions had the lowest coverages, while Nearctic, Palearctic and Oceanian had the highest. The Neotropical region had an intermediate coverage.”

should read:

“Oceanian, Afrotropical and Oriental biogeographic regions had the lowest coverages, while Nearctic, Neotropical and Palearctic had the highest. The Australian region had an intermediate coverage.”

In the Results section,

“The Afrotropical (18.61%), Australian (20.89%) and Oriental (21.25%) biogeographic regions had the lowest taxonomic coverage, while Nearctic (73.33%), Palearctic (48.53) and Oceanian (41.28%) had the highest (Fig. 1). The Neotropical region had an intermediate taxonomic coverage (34.15%).”

should read:

“Oceanian (5.67%), Afrotropical (16.89%) and Oriental (19.6%) biogeographic regions had the lowest taxonomic coverage, while Nearctic (64.7%), Neotropical (34.15%) and Palearctic (29.29%) had the highest (Fig. 1). The Australian region had an intermediate taxonomic coverage (20.89%).”

The original Article has been corrected.

