# Supplementary material for: Low coverage of species constrains the use of DNA barcoding to assess mosquito biodiversity
Source: Sci Rep. 2024 Mar 28;14:7432. doi: 10.1038/s41598-024-58071-1 (PMC10978826; doi:10.1038/s41598-024-58071-1)
Supplement: Supplementary file 5 — Supplementary Table 1. [file 41598_2024_58071_MOESM5_ESM.docx]

| markercode | Sequences |
| --- | --- |
| COI-5P | 50127 |
| COI-3P | 2669 |
| NA | 2651 |
|  | 2494 |
| ITS2 | 520 |
| 16S | 299 |
| COI-PSEUDO | 33 |
| COII | 33 |
| CYTB | 33 |
| COXIII | 32 |
| ND1 | 28 |
| ND3 | 28 |
| ND5-0 | 28 |
| ND6 | 28 |
| ND2 | 27 |
| ND4 | 27 |
| ND4L | 27 |
| 28S-D1-D2 | 8 |
| CQ11 | 5 |
| atp5 | 5 |
| 18S | 4 |
| 28S | 4 |
| ACE2 | 3 |

**Low coverage of species constrains the use of DNA barcoding to assess mosquito biodiversity.**

**Supplementary Table 1** – Markers found in the data set downloaded from Bold Systems.
