# Supplementary material for: Low coverage of species constrains the use of DNA barcoding to assess mosquito biodiversity
Source: Sci Rep. 2024 Mar 28;14:7432. doi: 10.1038/s41598-024-58071-1 (PMC10978826; doi:10.1038/s41598-024-58071-1)
Supplement: Supplementary file 6 — Supplementary Table 2. [file 41598_2024_58071_MOESM6_ESM.docx]

**Low coverage of species constrains the use of DNA barcoding to assess mosquito biodiversity.**

**Supplementary Table 2** – Models built to investigate taxonomic coverage/barcoding gap and results of the VIF tests.

| ##################### Results - COUNTRIES #################### |
| --- |
|  |
| > vif(f_aleatorio) |
| sequences richness endemicSp spMed |
| 1.799381 2.950131 2.595751 2.053707 |
|  |
| ##Modelo_1 had the best fit (lower AIC). |
|  |
| Generalized linear mixed model fit by maximum likelihood (Laplace Approximation) ['glmerMod'] |
| Family: binomial ( logit ) |
| Formula: cbind(speciesBold, speciesCulicidae) ~ sequences + richness + endemicSp + spMed + (1 \| biogeography) |
| Data: countryAnalysisUtD |
|  |
| AIC BIC logLik deviance df.resid |
| 1998.4 2016.1 -993.2 1986.4 136 |
|  |
| Scaled residuals: |
| Min 1Q Median 3Q Max |
| -5.115 -2.425 -1.120 1.127 16.503 |
|  |
| Random effects: |
| Groups Name Variance Std.Dev. |
| biogeography (Intercept) 2.353 1.534 |
| Number of obs: 142, groups: biogeography, 7 |
|  |
| Fixed effects: |
| Estimate Std. Error z value Pr(>\|z\|) |
| (Intercept) -2.51294 0.58336 -4.308 1.65e-05 *** |
| sequences 0.77770 0.04850 16.034 < 2e-16 *** |
| richness -0.28982 0.03855 -7.519 5.53e-14 *** |
| endemicSp 0.09832 0.02761 3.561 0.000369 *** |
| spMed -0.27357 0.04379 -6.248 4.16e-10 *** |
| --- |
| Signif. codes: 0 ‘***’ 0.001 ‘**’ 0.01 ‘*’ 0.05 ‘.’ 0.1 ‘ ’ 1 |
|  |
| Correlation of Fixed Effects: |
| (Intr) seqncs rchnss endmcS |
| sequences -0.077 |
| richness 0.002 0.104 |
| endemicSp 0.007 -0.354 -0.648 |
| spMed 0.010 -0.439 -0.467 0.151 |
|  |
| (2) coverage is higher in countries with low species richness and endemic richness; |
| (3) countries with a higher number of sequences also have a higher coverage; |
| (4) coverage is higher in countries with a higher number of medically important species. |
|  |
|  |
| ##################### Results - SPECIES #################### |
|  |
| vif(full_s) |
| sequences countriesBold medSp seqLength |
| 1.227827 1.222360 1.100654 1.002609 |
|  |
| > summary(m_4) |
|  |
| Call: |
| glm(formula = BLAST ~ sequences + countriesBold, family = binomial(link = "logit"), |
| data = spRep) |
|  |
| Deviance Residuals: |
| Min 1Q Median 3Q Max |
| -1.5589 -1.0979 0.8390 0.8561 3.2743 |
|  |
| Coefficients: |
| Estimate Std. Error z value Pr(>\|z\|) |
| (Intercept) -0.7319 0.1462 -5.006 5.57e-07 *** |
| sequences -4.5513 0.7563 -6.018 1.77e-09 *** |
| countriesBold -1.3877 0.2930 -4.737 2.17e-06 *** |
| --- |
| Signif. codes: 0 ‘***’ 0.001 ‘**’ 0.01 ‘*’ 0.05 ‘.’ 0.1 ‘ ’ 1 |
|  |
| (Dispersion parameter for binomial family taken to be 1) |
|  |
| Null deviance: 1308.1 on 944 degrees of freedom |
| Residual deviance: 1064.0 on 942 degrees of freedom |
| AIC: 1070 |
|  |
| Number of Fisher Scoring iterations: 8 |
|  |
| > summary(m_1) |
|  |
| Call: |
| glm(formula = BLAST ~ sequences + countriesBold + medSp, family = binomial(link = "logit"), |
| data = spRep) |
|  |
| Deviance Residuals: |
| Min 1Q Median 3Q Max |
| -1.5831 -1.0997 0.8405 0.8580 3.3167 |
|  |
| Coefficients: |
| Estimate Std. Error z value Pr(>\|z\|) |
| (Intercept) -0.7698 0.1568 -4.908 9.18e-07 *** |
| sequences -4.6420 0.7706 -6.024 1.70e-09 *** |
| countriesBold -1.4201 0.2969 -4.784 1.72e-06 *** |
| medSp1 0.2047 0.2936 0.697 0.486 |
| --- |
| Signif. codes: 0 ‘***’ 0.001 ‘**’ 0.01 ‘*’ 0.05 ‘.’ 0.1 ‘ ’ 1 |
|  |
| (Dispersion parameter for binomial family taken to be 1) |
|  |
| Null deviance: 1308.1 on 944 degrees of freedom |
| Residual deviance: 1063.5 on 941 degrees of freedom |
| AIC: 1071.5 |
|  |
| Number of Fisher Scoring iterations: 8 |
|  |
| > summary(m_2) |
|  |
| Call: |
| glm(formula = BLAST ~ sequences + countriesBold + seqLength, |
| family = binomial(link = "logit"), data = spRep) |
|  |
| Deviance Residuals: |
| Min 1Q Median 3Q Max |
| -1.6096 -1.0979 0.8009 0.8624 3.2792 |
|  |
| Coefficients: |
| Estimate Std. Error z value Pr(>\|z\|) |
| (Intercept) -0.72989 0.14603 -4.998 5.78e-07 *** |
| sequences -4.54701 0.75503 -6.022 1.72e-09 *** |
| countriesBold -1.38328 0.29302 -4.721 2.35e-06 *** |
| seqLength 0.02829 0.07627 0.371 0.711 |
| --- |
| Signif. codes: 0 ‘***’ 0.001 ‘**’ 0.01 ‘*’ 0.05 ‘.’ 0.1 ‘ ’ 1 |
|  |
| (Dispersion parameter for binomial family taken to be 1) |
|  |
| Null deviance: 1308.1 on 944 degrees of freedom |
| Residual deviance: 1063.9 on 941 degrees of freedom |
| AIC: 1071.9 |
|  |
| Number of Fisher Scoring iterations: 8 |
|  |
|  |
|  |
| ######## All tested models for Species |
|  |
|  |
| full_s <- glm(BLAST ~ sequences + countriesBold + medSp + seqLength, |
| family=binomial(link="logit"), data = spRep) |
|  |
| m_1 <- glm(BLAST ~ sequences + countriesBold + medSp, |
| family=binomial(link="logit"), data = spRep) |
|  |
| m_2 <- glm(BLAST ~ sequences + countriesBold + seqLength, |
| family=binomial(link="logit"), data = spRep) |
|  |
| m_3 <- glm(BLAST ~ sequences + medSp + seqLength, |
| family=binomial(link="logit"), data = spRep) |
|  |
| m_4 <- glm(BLAST ~ sequences + countriesBold, |
| family=binomial(link="logit"), data = spRep) |
|  |
| m_5 <- glm(BLAST ~ sequences + medSp, |
| family=binomial(link="logit"), data = spRep) |
|  |
| m_6 <- glm(BLAST ~ sequences + seqLength, |
| family=binomial(link="logit"), data = spRep) |
|  |
| m_7 <- glm(BLAST ~ countriesBold + medSp + seqLength, |
| family=binomial(link="logit"), data = spRep) |
|  |
| m_8 <- glm(BLAST ~ countriesBold + medSp, |
| family=binomial(link="logit"), data = spRep) |
|  |
| m_9 <- glm(BLAST ~ countriesBold + seqLength, |
| family=binomial(link="logit"), data = spRep) |
|  |
| m_10 <- glm(BLAST ~ countriesBold, |
| family=binomial(link="logit"), data = spRep) |
|  |
| m_11 <- glm(BLAST ~ medSp + seqLength, |
| family=binomial(link="logit"), data = spRep) |
|  |
| m_12 <- glm(BLAST ~ coverage, |
| family=binomial(link="logit"), data = spRep) |
|  |
| m_13 <- glm(BLAST ~ countriesBold, |
| family=binomial(link="logit"), data = spRep) |
|  |
| m_14 <- glm(BLAST ~ medSp, |
| family=binomial(link="logit"), data = spRep) |
|  |
| m_15 <- glm(BLAST ~ seqLength, |
| family=binomial(link="logit"), data = spRep) |

| ######## Results for all tested models for Species | | | | |
| --- | --- | --- | --- | --- |
| models | AICc | dAICc | df | weight |
| m_4 | 1070,06326 | 0 | 3 | 0,490321995 |
| m_1 | 1071,59151 | 1,528250119 | 4 | 0,228363178 |
| m_2 | 1071,941403 | 1,878143085 | 4 | 0,191711333 |
| full_s | 1073,462654 | 3,399393702 | 5 | 0,089600908 |
| m_6 | 1095,99078 | 25,92752056 | 3 | 1,15E-06 |
| m_5 | 1096,249826 | 26,18656615 | 3 | 1,01E-06 |
| m_3 | 1097,969867 | 27,90660757 | 4 | 4,27E-07 |
| m_8 | 1140,912722 | 70,84946173 | 3 | 2,02E-16 |
| m_10 | 1142,526057 | 72,46279757 | 2 | 9,02E-17 |
| m_13 | 1142,526057 | 72,46279757 | 2 | 9,02E-17 |
| m_7 | 1142,8022 | 72,73893961 | 4 | 7,86E-17 |
| m_9 | 1144,390585 | 74,32732476 | 3 | 3,55E-17 |
| m_14 | 1270,305988 | 200,2427286 | 2 | 1,62E-44 |
| m_11 | 1271,788245 | 201,7249853 | 3 | 7,70E-45 |
| m_15 | 1311,51385 | 241,4505903 | 2 | 1,82E-53 |
| m_12 | 1312,055317 | 241,9920568 | 2 | 1,39E-53 |
