# Supplementary material for: Low coverage of species constrains the use of DNA barcoding to assess mosquito biodiversity
Source: Sci Rep. 2024 Mar 28;14:7432. doi: 10.1038/s41598-024-58071-1 (PMC10978826; doi:10.1038/s41598-024-58071-1)
Supplement: Supplementary file 7 — Supplementary Table 3. [file 41598_2024_58071_MOESM7_ESM.docx]

**Low coverage of species constrains the use of DNA barcoding to assess mosquito biodiversity.**

**Supplementary Table 3** – key-words used in the queries for COI and ITS2.

| COI[GENE] OR COX1[GENE] OR CO1[GENE] OR |
| --- |
| MT-COI[GENE] OR COXI[GENE] OR MT-CO1[GENE] OR |
| Cytochrome c oxidase subunit I[GENE] OR |
| Cytochrome c oxidase subunit 1[GENE] OR |
| Cytochrome c oxidase I[GENE] OR |
| Cytochrome c oxidase 1[GENE] OR |
| COI-5P[GENE] OR CO1-5P |
|  |
| ITS2[ALL] OR ITSII[ALL] OR |
| internal transcribed spacer 2[ALL] OR |
| internal transcribed spacer II[ALL] |
